# Long-Term Offspring Outcomes Following Parental Exposure to BCR::ABL1 Tyrosine Kinase Inhibitors in Chronic Myeloid Leukemia: A Retrospective Cohort Study with Follow-Up to 24.6 Years

**DOI:** 10.3390/cancers18172885

**Published:** 2026-09-06

**Authors:** Sándor Pál, Margit Solymár, Hussain Alizadeh

**Affiliations:** 1 Department of Laboratory Medicine, University of Pécs Medical School, 7622 Pécs, Hungary; pal.sandor@pte.hu (S.P.);; 2 1st Department of Medicine, University of Pécs Medical School, 7622 Pécs, Hungary

**Keywords:** chronic myeloid leukemia, pregnancy, tyrosine kinase inhibitors, imatinib, nilotinib, dasatinib, congenital anomalies, offspring development, long-term follow-up

## Abstract

**Background**: Evidence regarding long-term outcomes among offspring following maternal or paternal exposure to BCR::ABL1 tyrosine kinase inhibitors (TKIs) is limited. We described pregnancy outcomes and offspring health through adolescence and early adulthood in a single-centre cohort of patients with chronic myeloid leukemia (CML). **Methods**: This retrospective, descriptive cohort study included patients with chronic-phase CML who experienced a pregnancy, or whose partner experienced a pregnancy, in association with TKI exposure and for whom longitudinal offspring follow-up was available. Long-term offspring outcomes were ascertained from detailed obstetric and pediatric medical records supplemented by parental reports. Fourteen patients (nine women and five men) contributed 21 pregnancy events and 23 live-born offspring, including two twin pregnancies in the paternal-exposure group. **Results**: In the maternal-exposure group, median gestational age at delivery was 38.0 weeks (range, 35–40), and four of 15 deliveries were preterm. In the paternal-exposure group, median gestational age was 39.0 weeks (range, 38–41), with no preterm deliveries. Median offspring follow-up was 252 months (range, 212–295), extending through adolescence and into early adulthood. One offspring had a primum atrial septal defect that was surgically corrected. No additional clinically documented major abnormalities in growth or developmental progress were identified during available follow-up. Given the small cohort, these observations were interpreted descriptively and were not considered evidence of equivalence with population-level rates. **Conclusions**: This small retrospective cohort provides unusually long observational follow-up of offspring after selected maternal or paternal TKI exposures. The findings are clinically informative but cannot establish reproductive safety or exclude rare adverse outcomes. Larger multicentre registries using standardized long-term developmental, neurocognitive, and endocrine assessments are needed.

## Simple Summary

BCR::ABL1 tyrosine kinase inhibitors are effective treatments for chronic myeloid leukemia, but information about the long-term health of children born after parental exposure to these medicines remains limited. We retrospectively evaluated 21 pregnancies and 23 live-born offspring of 14 patients with chronic myeloid leukemia. Exposure involved the mother in 15 pregnancies and the father in six pregnancies. The children were followed for a median of 21 years and up to 24.6 years. One child had an atrial septal defect that was surgically corrected. No additional major clinically documented abnormalities in growth or development were identified in the available medical records and parental reports. However, the cohort was small, lacked an unexposed comparison group, and did not undergo strict standardized long-term testing. Larger prospective studies with standardized assessments are needed.

## 1. Introduction

Chronic myeloid leukemia (CML) is driven by the BCR::ABL1 fusion gene generated by the reciprocal translocation t(9;22)(q34;q11), resulting in the Philadelphia chromosome [1,2,3]. The constitutively active BCR::ABL1 tyrosine kinase promotes uncontrolled proliferation, genomic instability, and resistance to apoptosis within hematopoietic stem and progenitor cells. BCR::ABL1 tyrosine kinase inhibitors selectively target the ATP-binding site of the fusion protein, thereby suppressing aberrant downstream signaling pathways including RAS/MAPK, PI3K/AKT, and JAK/STAT. This results in the restoration of normal hematopoiesis and has fundamentally changed the long-term prognosis of CML [3,4].

The introduction of BCR::ABL1-targeted tyrosine kinase inhibitors (TKIs) has transformed chronic myeloid leukemia (CML) from a life-threatening malignancy into a chronic condition associated with near-normal life expectancy [1,2,3,4]. Since the approval of imatinib in 2001, annual CML mortality has fallen from 10 to 20% to 1–2%, and CML-specific mortality is now 0.5–1%, with the result that prevalence in the United States alone is projected to reach 400,000–450,000 cases by 2040–2050 [3]. As survival has improved, quality of life, fertility preservation, and family planning have moved from peripheral concerns to central elements of routine practice [3,4,5,6,7,8,9]. Population-based European registries report a crude annual incidence of 0.7–1.0 per 100,000, a median age at diagnosis of 57–60 years, and a male-to-female ratio of 1.2–1.7:1, whereas contemporary North American data describe median ages as low as 48 years [10,11]. Global Burden of Disease analyses confirm a declining age-standardized burden overall but persistent disparities across sociodemographic strata, with the Middle East and North Africa among the regions carrying the highest disability-adjusted life-year rates [10,11]. Patients from Gulf Cooperation Council countries are frequently diagnosed at a younger age, and cultural and religious values may influence reproductive decisions, underscoring the need for culturally sensitive management [12,13].

CML in pregnancy remains uncommon, with an estimated incidence of approximately 1 per 100,000 pregnancies, yet it poses a distinctive challenge because therapeutic decisions must simultaneously address maternal disease control and fetal safety [14,15,16,17,18,19]. Fetal safety concerns arise from the fact that TKIs inhibit not only BCR::ABL1 but also PDGFR, VEGFR, and c-KIT signaling, all of which are essential for organogenesis [18,20]. First-trimester exposure has been associated with skeletal malformations, renal agenesis and hypoplasia, cardiac defects, neural tube abnormalities, and abdominal wall defects [21]. The largest early series of 180 imatinib-exposed pregnancies identified abnormalities in 12 infants, three of whom shared strikingly similar complex malformations involving the skeleton, kidneys, and exomphalos [21]. Preclinical studies reinforce these concerns: long-term imatinib in murine models produced epigenetic changes at imprinted placental genes and reduced placental vasculature that persisted after discontinuation, while in zebrafish, imatinib caused irreversible suppression of folliculogenesis and downregulation of VEGFAA and IGF2a [20]. However, evidence is not uniformly adverse; in a separate leukaemic mouse model, imatinib at therapeutic doses produced no histologic effect on folliculogenesis or spermatogenesis [20].

Among individual agents, imatinib has the most extensive human experience. The 2026 ASCO Management of Cancer During Pregnancy guideline notes that most imatinib-exposed pregnancies do not result in severe malformations but advises against first-trimester use unless no alternatives exist [14]. The Society for Maternal-Fetal Medicine (SMFM) states that second- and third-trimester imatinib data have been reassuring [15]. Nilotinib appears to have limited placental transfer, with fetal-to-maternal concentration ratios of approximately 0.5–0.58, and may carry lower teratogenic risk when used after organogenesis [7,20]. Dasatinib is fundamentally different and is strongly contraindicated at all stages of pregnancy because of its high placental transfer and documented associations with hydrops fetalis, fetal growth restriction, and intrauterine death [18,19,20,22]. In a disproportionality analysis of the WHO VigiBase encompassing 969 TKI-exposed pregnancy reports, hydrops fetalis (ROR 27; 95% CI, 5.4–130) and polyhydramnios (ROR 13; 95% CI, 3.3–54) were driven specifically by dasatinib [23]. Reproductive data for bosutinib, ponatinib, and asciminib remain insufficient for any recommendation, with product labeling documenting embryo-fetal toxicity at or below human therapeutic exposures [18,19,24,25,26].

Evidence regarding paternal TKI exposure is comparatively reassuring. A systematic review of 428 pregnancies fathered by men on uninterrupted therapy reported a 2.5% congenital malformation rate, comparable to background, and current NCCN guidelines state that TKI therapy need not be discontinued in male patients planning a pregnancy [16,27,28,29,30]. Treatment-free remission (TFR) has fundamentally changed reproductive counseling for women by enabling planned conception without ongoing TKI exposure [5,7,8,9]. In the final EURO-SKI analysis, 61% of patients maintained major molecular response (MMR) at six months and 46% at 36 months after discontinuation [8]. In a Japanese nationwide survey, patients fulfilling European LeukemiaNet discontinuation criteria achieved a TFR rate of 77% during pregnancy, but the median interval from diagnosis to conception was 10.6 years for planned pregnancies versus 4.1 years for unplanned ones, emphasizing that reproductive planning must begin at diagnosis [31]. If treatment is required during gestation, ASCO guidelines recommend that interferon-α may be administered in any trimester (Evidence quality: Moderate; Strength: Conditional), supported by its negligible placental transfer [14,15]. NCCN guidelines recommend that TKI therapy be stopped before natural conception, that fertility preservation be discussed before treatment initiation, and that patients be referred to a CML specialty center and high-risk obstetrician [16].

Despite this substantial evidence base, existing data remain focused on conception, fetal development, and neonatal outcomes [5,6,17,18,19,20,21,22,23,24,25,26,31]. Long-term childhood, adolescent, and early adult outcomes following parental TKI exposure are scarce; the methodological standard is set by prospective cohorts of children exposed in utero to maternal cancer, which have now followed offspring through ages 9, 12, and 15 using validated instruments. ASCO guidelines note that long-term follow-up into adulthood is lacking, particularly for fertility outcomes, and recommend continued surveillance [14,18,19]. To address this gap, we evaluated pregnancy outcomes and long-term offspring outcomes among children born to individuals with chronic-phase CML who conceived during TKI treatment.

The cohort encompasses both maternal and paternal exposures, includes pregnancies associated with imatinib, nilotinib, and dasatinib, and spans management strategies including treatment discontinuation, TFR, interferon therapy, and selective later-gestation TKI use, with follow-up extending to 24.6 years.

## 2. Materials and Methods

This retrospective, single-centre, descriptive cohort study evaluated pregnancy outcomes and long-term health information among live-born offspring following maternal or paternal tyrosine kinase inhibitor (TKI) exposure in patients with chronic myeloid leukemia (CML). The source population comprised 105 consecutive patients initially diagnosed with chronic-phase CML, including 49 women and 56 men, who were treated with TKIs at Tawam Hospital, Al Ain, United Arab Emirates, between June 2001 and December 2009.

Among the 105 patients constituting the source population, 91 did not contribute eligible pregnancy events during the observation period. Fourteen patients fulfilled the study inclusion criteria and contributed a total of 21 pregnancy events. No identified pregnancy event was excluded because of unavailable offspring follow-up.

Pregnancy events were ascertained during longitudinal clinical follow-up from 20 June 2001 to 1 June 2026. Pregnancies among female patients were identified from clinical and obstetric documentation. Partner pregnancies among male patients were identified from reproductive histories recorded during routine hematology follow-up and were verified against available obstetric or offspring records where possible. Pregnancy ascertainment was not based on a dedicated prospective registry. Therefore, unreported partner pregnancies and very early or otherwise clinically unrecognized pregnancy losses may not have been captured.

Patients were included if they experienced a pregnancy, or had a partner who experienced a pregnancy, in association with TKI exposure, and long-term health information was available for the resulting offspring. Cohort entry was defined as the date of confirmed pregnancy or partner pregnancy. Maternal exposure was defined as maternal TKI use at conception or during pregnancy, whereas paternal exposure was defined as paternal TKI use at the time of conception.

Detailed exposure timing information was available for all maternal pregnancies and was abstracted from contemporaneous clinical records. Most maternal pregnancies involved continuous TKI exposure throughout gestation and lactation. Exceptions included one pregnancy with brief interferon-alpha exposure during early gestation before transition to imatinib because of treatment intolerance, as well as one pregnancy in which imatinib was temporarily interrupted for approximately four weeks before being reinitiated because of progressive leukocytosis, and one pregnancy managed using sequential interferon-alpha, hydroxycarbamide, leukapheresis, and imatinib therapy. These treatment patterns are summarized in Supplementary Table S1. Because individual patients could contribute more than one pregnancy, pregnancy-level observations were not considered statistically independent.

Reporting follows the Strengthening the Reporting of Observational Studies in Epidemiology (STROBE) guidance.

Pregnancy events were identified during routine clinical follow-up. Initial home urinary pregnancy testing was followed by serum human chorionic gonadotropin testing and subsequent ultrasonographic confirmation. A total of 21 pregnancy events were identified, resulting in 23 live-born offspring, including two twin pregnancies. No miscarriage, elective termination, stillbirth, or neonatal death was documented among the identified pregnancy events. No pregnancy event was excluded because of unavailable offspring follow-up. The study was approved by the Regional Ethics Committee, and written informed consent was obtained from all participants.

Treatment response was classified using the European LeukemiaNet criteria applicable during follow-up. Complete hematologic response (CHR), complete cytogenetic response (CCyR), and major molecular response (MMR) were abstracted from routine clinical records. Hematologic assessments were performed monthly until CHR and approximately every three months thereafter. BCR::ABL1 transcript levels were measured using quantitative real-time polymerase chain reaction and reported on the International Scale where available.

Long-term offspring information was obtained from detailed obstetric medical records and pediatric documentation and was supplemented by detailed parental reports. Outcomes of interest included survival, congenital anomalies, documented medical conditions, physical growth, age-appropriate developmental milestones, educational progress where available, and clinically recognized cognitive or developmental disorders. Information from parental reports was considered complementary to the available medical documentation. Neurocognitive outcomes were reviewed together with developmental and educational progress where available. Growth information included weight, height, and head circumference. Vital status, growth-related information, developmental information, educational outcomes, and endocrine information were available for all 23 identified live-born offspring through review of available medical records supplemented by detailed parental interviews. The completeness of offspring follow-up according to outcome domain and data source is summarized in Supplementary Table S2.

Follow-up extended through adolescence and early adulthood, with a maximum documented duration of 295 months (24.6 years). However, because of the retrospective nature of the study, the completeness and level of detail of individual growth, developmental, educational, and health-related variables varied according to the availability of historical medical records and parental information. Long-term follow-up included systematic review of pediatric and adult medical records, developmental progress, educational outcomes, endocrine information where available, fertility-related information where available, and detailed parental interviews. However, because of the retrospective design and prolonged observation period, identical protocol-driven assessment using the same validated neuropsychological, endocrine, and reproductive instruments was not available across the entire cohort. Consequently, subtle abnormalities requiring specialized formal testing cannot be completely excluded.

The final data lock was 1 June 2026. Because of the retrospective descriptive design and the rarity of eligible pregnancy events, no formal sample-size calculation was performed. The study included all consecutive eligible pregnancy events identified within the source population during the study period. Analyses were descriptive and exploratory. Patient-, pregnancy-, and offspring-level characteristics are reported separately. Continuous variables are summarized using medians and ranges, and categorical variables using counts and percentages. The observed congenital anomaly frequency is reported as a descriptive proportion; no equivalence or non-inferiority inference relative to population rates was intended. Owing to the small cohort, repeated pregnancies contributed by the same patient, twin gestations, and limited statistical power for rare outcomes, comparative hypothesis tests were not used to support safety conclusions. Because of the small cohort, the rarity of the clinical setting, and the distinctive combinations of treatment and pregnancy characteristics, detailed de-identified pregnancy-level data are provided in Supplementary Table S1; however, exact dates and potentially identifying information were omitted to reduce re-identification risk. Analyses were performed using Python version 3.13 and SciPy (version 1.18.0).

## 3. Results

**Figure 1:**
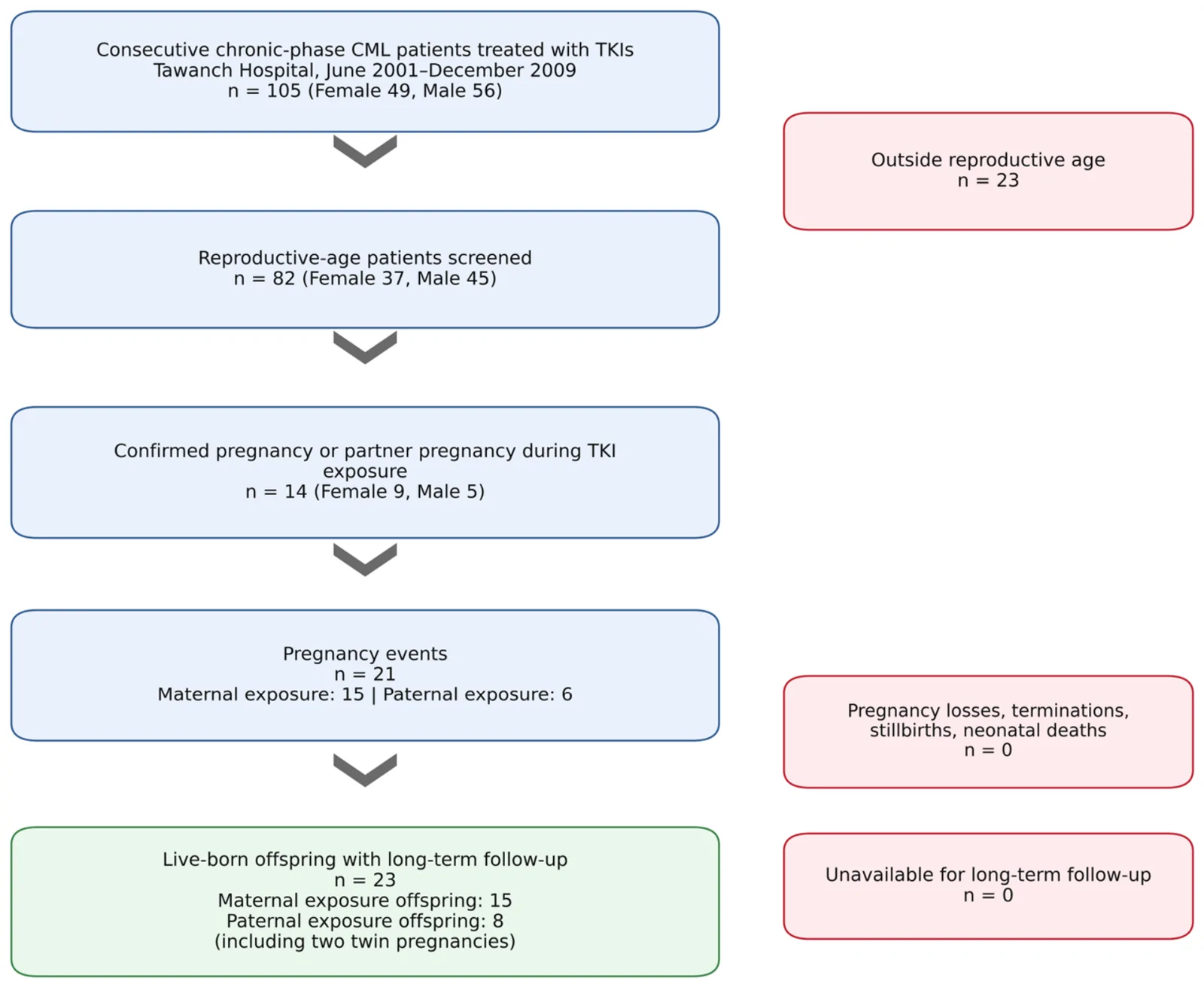
Study flow diagram. Consecutive chronic-phase chronic myeloid leukemia (CML) patients treated with tyrosine kinase inhibitors (TKIs) were screened for pregnancy events. Pregnancy outcomes and long-term follow-up of live-born offspring are shown. No pregnancy events were excluded because of unavailable follow-up.

Patient selection and pregnancy outcomes are summarized in Figure 1. The study cohort was derived from a source population of 105 consecutive chronic-phase CML patients treated during the study period. Fourteen patients fulfilled the eligibility criteria and contributed 21 documented pregnancy events. No eligible pregnancy was excluded from analysis because of unavailable long-term offspring follow-up.

Fourteen patients with chronic-phase CML met the study eligibility criteria, including nine women who contributed 15 pregnancies and five men whose partners contributed six pregnancies. These 21 pregnancies resulted in 23 live-born offspring because two paternal-exposure pregnancies were twin gestations. No miscarriage, elective termination, stillbirth, or neonatal death was documented among the 21 identified pregnancy events, and no pregnancy event was excluded because of unavailable offspring follow-up.

Patient, pregnancy, treatment, and offspring characteristics according to parental exposure group are summarized in Table 1. Median maternal age at pregnancy was 34.0 years (range, 22–47).

Among the nine female patients, 15 pregnancies were included. Six pregnancies (40.0%) involved imatinib exposure throughout gestation, and five (33.3%) involved initial interferon-alpha treatment followed by imatinib. Exposure timing information was available for all maternal pregnancies. In five pregnancies, interferon-alpha was administered briefly during early gestation before transition to imatinib. In one pregnancy, imatinib was temporarily discontinued for approximately four weeks but was subsequently restarted because of progressive leukocytosis and continued thereafter throughout pregnancy. One additional pregnancy involved sequential interferon-alpha, hydroxycarbamide, leukapheresis, and imatinib treatment for maternal disease control.

In one pregnancy, the patient was off treatment at conception and restarted imatinib during the second trimester. One pregnancy involved sequential interferon-alpha, hydroxycarbamide, leukapheresis, and imatinib; one involved nilotinib throughout gestation; and one involved dasatinib throughout gestation. At conception, 10 pregnancies (66.7%) occurred in patients with stable MMR or deeper response, two (13.3%) in CCyR, two (13.3%) in CHR, and one (6.7%) in a patient initially diagnosed with chronic-phase CML who was in accelerated phase while off treatment at conception and subsequently received imatinib dose escalation. Median gestational age at delivery was 38.0 weeks (range, 35–40), and four deliveries (26.7%) occurred before 37 weeks. The 15 included pregnancies resulted in 15 live-born offspring.

Five male patients contributed six partner pregnancies. At conception, imatinib was used in four pregnancies (66.7%), nilotinib in one (16.7%), and sequential or combined imatinib and nilotinib exposure was recorded in one (16.7%). All five men were in stable MMR at the time of conception. Median gestational age at delivery was 39.0 weeks (range, 38–41), and no delivery occurred before 37 weeks. Two pregnancies were twin pregnancies, resulting in eight live-born offspring.

**Figure 2:**
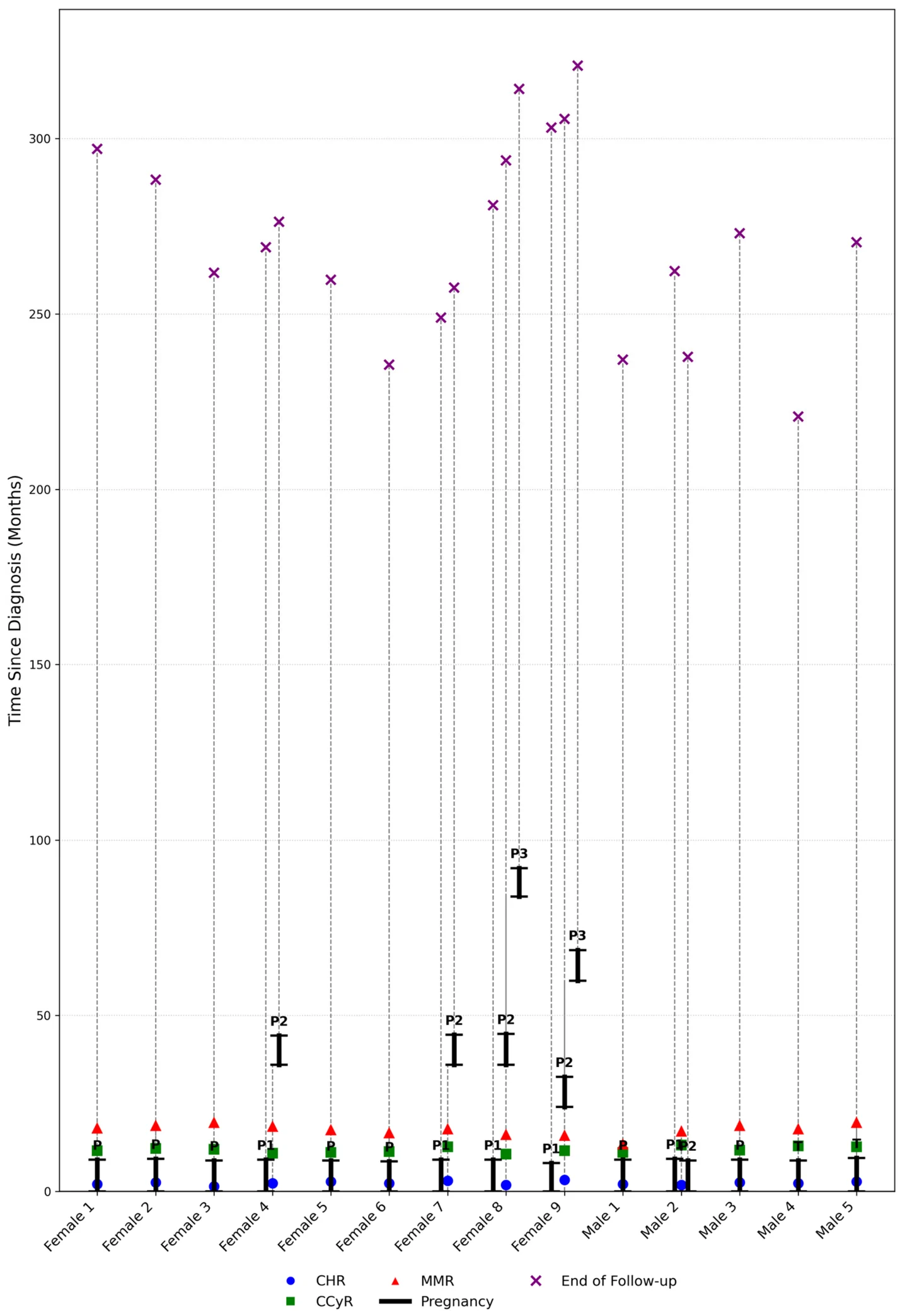
Long-term clinical timelines of patients with CML and their offspring. Individual timelines are shown from CML diagnosis or TKI initiation and include achievement of complete hematologic response (CHR), complete cytogenetic response (CCyR), and major molecular response (MMR), pregnancy windows, and duration of offspring follow-up. Offspring outcomes at the latest assessment are indicated at the end of each timeline. P1–P3 denote successive pregnancies.

The 23 offspring were followed for a median of 252 months (range, 212–295), corresponding to approximately 17.7–24.6 years. Median follow-up was 252 months (range, 213–295) in the maternal-exposure group and 241 months (range, 212–264) in the paternal-exposure group. These values are presented descriptively. Integrated patient, pregnancy, and offspring follow-up timelines are shown in Figure 2.

A detailed summary of follow-up completeness according to data source and outcome domain is provided in Supplementary Table S2.

One offspring in the maternal-exposure group was diagnosed with a primum atrial septal defect during early childhood. Maternal treatment during the pregnancy included several interventions for CML control, precluding attribution to any individual treatment. The defect was surgically corrected, and no major subsequent physical or cognitive abnormality was documented during long-term follow-up. Among the remaining 22 offspring, the available obstetric and pediatric medical records and detailed parental reports did not document major clinically recognized abnormalities in physical growth or developmental progress, including age-appropriate milestone attainment. No clinically recognized cognitive or developmental disorder was documented during the available follow-up. No inference of equivalence with population congenital anomaly rates was made because of the small cohort and statistical imprecision.

## 4. Discussion

This retrospective single-centre cohort provides long-term observational data on 23 offspring born after maternal or paternal TKI exposure in chronic-phase CML. Taken together, the present findings are broadly consistent with the available literature regarding both maternal and paternal exposure to BCR::ABL1 tyrosine kinase inhibitors. Contemporary ASCO, NCCN, and SMFM recommendations continue to discourage maternal TKI exposure during pregnancy, particularly during organogenesis, while acknowledging that clinical outcomes are heterogeneous and strongly influenced by timing of exposure. Similarly, published series evaluating paternal TKI exposure have generally reported reassuring pregnancy outcomes without evidence of a major increase in congenital anomalies. Importantly, however, nearly all available studies focus on pregnancy, neonatal outcomes, or early childhood. In contrast, information extending into adolescence and adulthood remains extremely limited. The present cohort therefore complements the existing literature by providing unusually long observational follow-up extending beyond two decades, while simultaneously reinforcing the need for continued prospective surveillance and standardized long-term assessment [14,15,16,17,18,19,27,28,29,30].

The principal contribution of the present study is the unprecedented duration of offspring follow-up, extending into adolescence and early adulthood, with a median follow-up of 252 months and a maximum follow-up of 295 months (24.6 years), well beyond the neonatal and childhood periods that dominate the existing literature.

A single congenital cardiac anomaly, consisting of a surgically corrected primum atrial septal defect, was observed. These observations should be regarded as hypothesis-generating and must not be interpreted as evidence of reproductive safety; current guideline recommendations continue to state that conception while on active TKI therapy is strongly discouraged [14,15,16,18,19,20].

The maternal cohort was heterogeneous with respect to treatment pattern, exposure timing, and disease-response state. Four of 15 deliveries were preterm, a proportion that must be interpreted against relevant comparator populations rather than attributed to TKI exposure. In a Swedish nationwide study of 342 pregnancies in women with myeloproliferative neoplasms, preterm birth occurred in 14% versus 4% of matched controls [32]. Among women with a prior history of leukemia or lymphoma, preterm birth prevalence was 14.6% in California and 12.0% in Iowa, compared with 7.8% and 8.2% in women without a cancer history (adjusted odds ratios 1.89 and 1.61, respectively) [33]. When a hematological malignancy is active during gestation, the effect is far larger: in a French nationwide cohort of nearly 10 million pregnancies, preterm birth occurred in 35.4% versus 5.4% in the reference group (adjusted odds ratio 11.76) [34]. In a cohort of this size, without an unexposed comparison group, the contributions of the underlying disease, maternal characteristics, obstetric factors, and treatment cannot be disentangled. Accordingly, the observed preterm delivery frequency in the present cohort falls within the broad range previously reported among women affected by hematologic malignancies, although definitive causal attribution to TKI exposure remains impossible.

Similarly, one congenital anomaly was observed among 23 offspring (4.3%; exact 95% CI, approximately 0.1–21.9%), which is not statistically distinguishable from background population rates, with congenital heart defects—the most common category—occurring at a EUROCAT prevalence of 6.5 per 1000 births. The anomaly followed multiple maternal interventions, including imatinib from week 13, making causal attribution impossible.

Published evidence continues to support avoidance of TKI exposure during organogenesis and avoidance of dasatinib throughout pregnancy. In the VigiBase disproportionality analysis, overreported outcomes with TKIs as a class included hydrops fetalis (ROR 13; 95% CI, 1.5–110) and polyhydramnios (ROR 5; 95% CI, 1.3–20), with molecule-specific signals driven by dasatinib (hydrops fetalis ROR 27; 95% CI, 5.4–130) [23]. ASCO and SMFM guidelines both classify dasatinib as strongly contraindicated at all gestational stages [14,15]. The single favorable outcome observed after dasatinib exposure should be regarded solely as an anecdotal observation. It does not modify current recommendations and should not be interpreted as evidence supporting the safety of dasatinib during pregnancy. The observation that later-gestation imatinib was tolerated is consistent with existing data from ASCO and SMFM guidelines indicating reassuring second- and third-trimester outcomes, and with a UK series of 22 cases of CML diagnosed in pregnancy in which 21 healthy babies were delivered without treatment-related fetal abnormalities [17,31]. Equally relevant is growing evidence that brief inadvertent first-trimester exposure followed by prompt discontinuation may carry lower risk than historically assumed. In a Chinese cohort of 59 pregnancies with a median TKI exposure of four weeks, all pregnancies resulted in normal childbirth without birth abnormalities, and the median times to regain major molecular response and deep molecular response after therapy resumption were 3 and 6 months, respectively [35]. These findings support the counseling position that discovery of an unplanned pregnancy during TKI therapy is not in itself an indication for termination.

Loss of molecular response during pregnancy-related treatment interruption is common but usually recoverable. In a multicentre case–cohort of 16 women, 11 lost MMR and two lost complete hematological response during pregnancy, yet 14 re-established MMR on therapy resumption; the depth of response before conception, rather than its duration, predicted restoration of control. In 17 planned pregnancies, achieving MR4 or MR4.5 and an MMR duration of at least 3.5 years were significantly associated with longer failure-free survival during interruption. Pregnancy itself does not appear to compromise long-term disease control: in a comparative cohort, 10-year progression-free survival was 100% among compliant women with unplanned pregnancy versus 72.7% among noncompliant women, indicating that adherence after interruption drives outcome [5,6,17].

The paternal-exposure group comprised six pregnancies and eight offspring, including two sets of twins, with no anomaly or preterm delivery documented. These observations are consistent with the systematic review reporting 428 pregnancies from 374 fathers on uninterrupted TKI therapy, with 93.5% live births and a 2.5% malformation rate, and with NCCN guidance that TKI need not be discontinued for paternal conception [16,27,28,29,30]. The subgroup is nevertheless too small to quantify risk or exclude uncommon outcomes. Because pregnancy events were identified from routine clinical follow-up, early spontaneous miscarriages occurring before medical recognition may not have been captured. Consequently, the apparent absence of pregnancy losses should not be interpreted as evidence that parental TKI exposure does not increase the risk of early pregnancy loss.

The standard against which offspring follow-up should be judged is set by prospective multicentre cohorts: at age 9, 151 children exposed in utero to maternal cancer had cognitive outcomes within normal ranges, with Full Scale IQ rising 1.6 points per additional gestational week; at ages 12 and 15, 166 adolescents had normal neurocognition, cardiac function, and pubertal development [36], with vulnerabilities attributable to prematurity rather than treatment. Critically, formal testing at age 6 in a third cohort detected statistically lower mean verbal IQ (98.1 vs. 104.4, *p* = 0.001) in exposed children despite scores within normal ranges—precisely the effect magnitude that questionnaire-based ascertainment cannot detect. At the population level, a Danish registry of 2,526,163 liveborn children, including 690 exposed to maternal cancer in utero, found no excess mortality (adjusted hazard ratio 0.8; 95% CI, 0.4–1.5) or increased congenital, somatic, or psychiatric disease [37]. There is also specific reason to consider endocrine and growth endpoints: a systematic review of 14 studies in children receiving TKIs directly documented reduced height z-scores, decreased IGF-1, and final height below mid-parental target [38], while an independent FAERS analysis identified disproportionate signals for pubertal failure with imatinib [39]. Because tyrosine kinases participate in growth hormone and IGF-1 signaling, these endpoints are biologically plausible targets of in utero exposure and warrant protocolized assessment.

The most actionable clinical message from the present data concerns timing. In the Japanese nationwide survey, the median interval from CML diagnosis to conception was 10.6 years for planned pregnancies versus 4.1 years for unplanned ones, and patients fulfilling ELN discontinuation criteria achieved a TFR rate of 77% during pregnancy [31]. This reinforces the recommendation that reproductive planning begin at diagnosis. NCCN now explicitly identifies young patients whose goal is deep molecular response with eventual discontinuation for family planning as a subgroup in whom second-generation or allosteric TKIs should be considered [16].

### 4.1. Strengths and Limitations

The principal strengths of this study are the unusually long observation period, inclusion of both maternal and paternal exposure, and availability of long-term clinical information for all identified live-born offspring. The present findings may assist counselling of patients with CML who conceive during TKI treatment or who present with an unplanned pregnancy. While current recommendations to avoid maternal TKI exposure during pregnancy remain unchanged, the prolonged follow-up reported here provides clinically informative observational data regarding long-term offspring health after selected maternal and paternal exposures. These data may contribute to shared decision-making when balancing maternal disease control against potential fetal risk. To our knowledge, this represents one of the longest reported follow-up durations for offspring born following parental TKI exposure.

Important limitations include the retrospective single-centre design, small sample size, potential selection and survivorship bias, uncertain completeness of pregnancy-event ascertainment, repeated pregnancies contributed by individual patients, twin gestations, and the absence of an internal comparison group. Early pregnancy losses and elective terminations may have been underascertained. The absence of an internal unexposed comparator group, such as offspring conceived during treatment-free remission or healthy sibling controls, precludes estimation of relative risk, demonstration of equivalence with background population outcomes, or conclusions regarding reproductive safety.

Long-term follow-up information was available for all identified live-born offspring. Outcomes were ascertained retrospectively from available obstetric, pediatric, and adult medical records supplemented by structured parental interviews. However, the completeness and level of detail of the available documentation varied across offspring and outcome domains, and parental reporting may have introduced recall bias. Uniform prospective assessments using validated neurodevelopmental, neurocognitive, endocrine, reproductive, or psychosocial instruments were not performed. Consequently, subtle or domain-specific abnormalities requiring formal assessment may have remained undetected.

With 23 offspring, the upper 95% confidence bound around the single observed congenital anomaly encompasses rates well above population background levels. Findings should not be extrapolated to bosutinib, ponatinib, or asciminib, for which product labeling documents embryo-fetal toxicity at or below therapeutic exposures and human pregnancy data are confined to isolated case reports [18,19,24,25,26]. Because these agents are increasingly used in younger patients who may pursue pregnancy, the applicability of an imatinib-, nilotinib-, and dasatinib-based cohort to contemporary practice may progressively diminish.

### 4.2. Clinical Implications

The present findings may assist counselling of patients with chronic myeloid leukemia who conceive despite TKI exposure or who present with an unplanned pregnancy. While current recommendations to avoid TKI exposure during pregnancy remain unchanged, the prolonged follow-up reported here provides clinically informative observational data regarding long-term offspring health after selected parental exposures. These findings may contribute to shared decision-making when balancing maternal disease control against potential fetal risk. However, they should be regarded as hypothesis-generating and should not be interpreted as evidence of reproductive safety.

## 5. Conclusions

This retrospective cohort provides one of the longest reported observational follow-up periods of offspring born following parental exposure to BCR::ABL1 tyrosine kinase inhibitors in chronic myeloid leukemia, extending to 24.6 years. One congenital anomaly was documented among 23 offspring, and no additional major clinically documented abnormalities in growth or development were identified from the available medical records and parental reports during follow-up. These findings are clinically informative but do not establish reproductive safety and cannot exclude rare or subtle adverse outcomes. Larger prospective registries incorporating standardized long-term neurocognitive, endocrine, growth, and reproductive assessments are required to define the long-term consequences of parental TKI exposure more precisely.

## Figures and Tables

**Table 1:** Characteristics of patients, pregnancy events, treatment exposures, and live-born offspring according to parental exposure group. TKI-associated pregnancy categories were not mutually exclusive because some pregnancy events involved sequential or combined exposure to more than one TKI. Percentages may therefore sum to more than 100%. IFN, interferon-alpha; HC, hydroxycarbamide; MMR, major molecular response; TKI, tyrosine kinase inhibitor. * All five male patients were in stable MMR at conception; one patient contributed more than one pregnancy event.

Characteristic	Maternal Exposure	Paternal Exposure	Total Cohort
**Patients, n**	9	5	14
**Pregnancy events, n**	15	6	21
**Live-born offspring, n**	15	8	23
**Twin pregnancies, n**	0	2	2
**Parental age at conception, years (range—min–max)**	22–47	28–71	22–71
**Imatinib-associated pregnancies, n (%)**	13 (86.7)	5 (83.3)	18 (85.7)
**Nilotinib-associated pregnancies, n (%)**	1 (6.7)	2 (33.3)	3 (14.3)
**Dasatinib-associated pregnancies, n (%)**	1 (6.7)	0 (0.0)	1 (4.8)
**Parental treatment pattern, offspring-level distribution, n (%)**	(N = 15)	(N = 8)	(N = 23)
**—Imatinib only**	6 (40.0)	4 (50.0)	10 (43.5)
**—Alpha-interferon → Imatinib**	5 (33.3)	0 (0.0)	5 (21.7)
**—Nilotinib only**	1 (6.7)	2 (25.0)	3 (13.0)
**—Imatinib + Nilotinib**	0 (0.0)	2 (25.0)	2 (8.7)
**—Off-treatment → Imatinib**	1 (6.7)	0 (0.0)	1 (4.3)
**—IFN → HC → Leukapheresis → Imatinib**	1 (6.7)	0 (0.0)	1 (4.3)
**—Dasatinib only**	1 (6.7)	0 (0.0)	1 (4.3)
**MMR or deeper at conception, n (%)**	10 (66.7)	6 (100) *	16 (76.2)
**Gestational age, median (range), weeks**	38 (35–40)	39 (38–41)	38 (35–41)
**Preterm deliveries, n (%)**	4 (26.7)	0 (0.0)	4 (19.0)
**Documented pregnancy losses, n**	0	0	0
**Documented congenital anomalies, n/N**	1/15	0/8	1/23
**Offspring follow-up, median (range), months**	252 (213–295)	241 (212–264)	252 (212–295)

## Data Availability

Data are available upon reasonable request from the corresponding author following joint approval of the Tawam Hospital Research Review Committee, the FMHS Research Ethics Committee, and the Al Ain Regional District Ethics and Research Committee.

## Supplementary Materials

The following supporting information can be downloaded at https://www.mdpi.com/article/10.3390/cancers18172885/s1. Supplementary Table S1. De-identified pregnancy-level treatment exposure patterns and offspring outcomes. Supplementary Table S2. Completeness of offspring follow-up information according to data source and outcome domain (N = 23 offspring).

## Abbreviations

**Abbreviation**	**Full term**
ASCO	American Society of Clinical Oncology
ASD	Atrial septal defect
BCR::ABL1	Breakpoint cluster region::ABL proto-oncogene 1 fusion gene
CCyR	Complete cytogenetic response
CHR	Complete hematologic response
CI	Confidence interval
CML	Chronic myeloid leukemia
ELN	European LeukemiaNet
EUROCAT	European network of population-based registries for the epidemiological surveillance of congenital anomalies
EURO SKI	European Stop Tyrosine Kinase Inhibitor study
FAERS	FDA Adverse Event Reporting System
FDA	US Food and Drug Administration
FMHS	Faculty of Medicine and Health Sciences
GBD	Global Burden of Disease
GCC	Gulf Cooperation Council
HU	Hydroxyurea
IFN	Interferon
IGF-1	Insulin-like growth factor 1
IGF2a	Insulin-like growth factor 2a
IQ	Intelligence quotient
MMR	Major molecular response
MR4	Molecular response with a BCR::ABL1 level ≤0.01% on the International Scale
MR4.5	Molecular response with a BCR::ABL1 level ≤0.0032% on the International Scale
NCCN	National Comprehensive Cancer Network
PDGFR	Platelet-derived growth factor receptor
ROR	Reporting odds ratio
SMFM	Society for Maternal-Fetal Medicine
TFR	Treatment-free remission
TKI	Tyrosine kinase inhibitor
UAE	United Arab Emirates
UK	United Kingdom
US	United States
VEGFAA	Vascular endothelial growth factor Aa
VEGFR	Vascular endothelial growth factor receptor
WHO	World Health Organization
