# Supplementary material for: Long-Term Offspring Outcomes Following Parental Exposure to BCR::ABL1 Tyrosine Kinase Inhibitors in Chronic Myeloid Leukemia: A Retrospective Cohort Study with Follow-Up to 24.6 Years
Source: Cancers (Basel). 2026 Sep 6;18(17):2885. doi: 10.3390/cancers18172885 (PMC13565506; doi:10.3390/cancers18172885)
Supplement: Supplementary file 1 [file cancers-18-02885-s001.zip › cancers-4528941-supplementary.pdf]

Supplementary Table S1. De-identified pregnancy-level treatment exposure patterns and offspring outcomes

Pregnancy ID	Exposure Type	Treatment Pattern During Pregnancy	Pregnancy Outcome	Long-Term Offspring Outcome
**M1-M6**	Maternal	Imatinib throughout gestation and lactation	Live birth	No clinically documented major abnormality
**M7-M11**	Maternal	Brief IFN-α exposure during early gestation followed by imatinib throughout the remainder of pregnancy and lactation	Live birth	No clinically documented major abnormality
**M12**	Maternal	Temporary imatinib interruption (~4 weeks) during gestation followed by re-initiation because of progressive leukocytosis; continued thereafter throughout pregnancy and lactation	Live birth	No clinically documented major abnormality
**M13**	Maternal	Sequential disease-control strategy: IFN-α → hydroxycarbamide → leukapheresis → imatinib	Live birth	Primum atrial septal defect; surgically corrected
**M14**	Maternal	Nilotinib throughout gestation and lactation	Live birth	No clinically documented major abnormality
**M15**	Maternal	Dasatinib throughout gestation and lactation	Live birth	No clinically documented major abnormality
**P1-P4**	Paternal	Imatinib exposure at conception	Live birth	No clinically documented major abnormality
**P5**	Paternal	Nilotinib exposure at conception	Live birth	No clinically documented major abnormality
**P6**	Paternal	Imatinib and/or nilotinib exposure at conception	Twin live birth	No clinically documented major abnormality

Note. Abbreviations: IFN-α, interferon-alpha.

Pregnancy identifiers were created solely for reporting purposes and do not correspond to patient identifiers. Exact dates and potentially identifying clinical information were omitted to preserve confidentiality. Exposure information was abstracted from contemporaneous medical records. Long-term offspring outcomes were based on available obstetric, pediatric, and adult medical records supplemented by parental reports.

Supplementary Table S2. Completeness of offspring follow-up information according to data source and outcome domain (N = 23 offspring)

Variable	Number (%)	Information source
Offspring with available pediatric medical records	23 (100)	Standardized assessment
Offspring with available adult medical records (where age-appropriate)	19 (82.6)	Standardized assessment
Offspring with documented growth information (weight and/or height and/or head circumference)	23 (100)	Standardized assessment
Offspring with documented developmental information	23 (100)	Parental report
Offspring with educational progress information available	23 (100)	Parental report
Offspring with endocrine-related information available in medical records	23 (100)	Standardized assessment
Offspring with parental interview data	23 (100)	Standardized assessment
Offspring with follow-up based exclusively on parental report	0 (0)	Not applicable

Note: Outcome ascertainment was based on review of available pediatric and adult medical records supplemented by structured parental interviews. Growth information included recorded anthropometric data available in medical documentation. Developmental information referred to documented age-appropriate developmental progress, milestone attainment, or absence of clinically recognized developmental disorders in available records or parental reports. Educational information referred to school attendance and educational progress where available. Endocrine information referred to clinically documented endocrine history and available medical information during follow-up. Because of the retrospective design and prolonged observation period, the level of detail differed among offspring and outcome domains. Uniform prospective neurocognitive, reproductive, endocrine, psychosocial, and developmental assessments using validated standardized instruments were not performed. Consequently, subtle abnormalities requiring specialized testing cannot be excluded.
